# Knowledge, practice of cervical cancer screening and associated factors among women police members of Addis Ababa police commission Ethiopia

**DOI:** 10.1186/s12885-023-11478-x

**Published:** 2023-10-10

**Authors:** Tangut Misgun, Dereje Bayissa Demissie

**Affiliations:** 1 Santé Medical College School of Public Health Department of Reproductive Health, Addis Ababa, Ethiopia; 2 St. Paul’s Hospital Millennium Medical College, Addis Ababa, Ethiopia

**Keywords:** Cervical, Cancer, Screening, Knowledge, Practice

## Abstract

**Background:**

Cervical cancer is a public health problem. It is one of the leading causes of death in women worldwide and the second leading cause of female cancer-related deaths. Cervical cancer screening enables the detection of abnormal cervical cells, including precancerous cervical lesions, as well as early-stage cervical cancer. Routine cervical screening has been shown to reduce both the incidence and mortality of the disease.

**Objective:**

The aim of this study was to assess knowledge, practice of cervical cancer screening and associated factors among women police members at Addis Ababa police commission Ethiopia 2022.

**Method:**

An institutional-based cross-sectional study was conducted at the Addis Ababa police commission in the Lidet Sub-city police department from December 1st to January 30th, 2022. The data were collected through a structured, self-administered questionnaire from 361 randomly selected police officers. The collected data were analyzed using SPSS version 26 software in descriptive statistics, binary, and multivariable logistic regression analysis to identify factors associated with the outcome variable at PV = 0.05 with an AOR and 95% CI. Results: The complete response rate was 97.57% (361/371). This found that 59.5% of the total study participants were aged between 18 and 29 years old, and 47.3% were married in marital status. This study determined knowledge status of police commission towards cervical cancer screening revealed that 183(49.5%) had good knowledge, with identified factors which increases likelihood of good knowledge were attitude [AOR = 2.03, 95%CI;( 1.25–3.3)] and cervical cancer screening practice [AOR = 2.0, 95%CI (1.15–3.53)] respectively.

This is determined the prevalence of cervical cancer screening practice was 68(18.4%)with 95% CI(14.3–22.4) with identified factors which increases likelihood of cervical cancer screening practice were age, [AOR = 3.24, 95% CI;( 1.08–9.75)], marital status [AOR = 3.88,95%CI,(1.55–9.73)] monthly income [AOR = 4.82,95%CI;(1.44–16.12)],religion[AOR = 8.65,95%CI,( 1.65–45.46)] and knowledge [AOR = 2.35,95%CI;( 1.22–4.52)] respectively. The main reason reported for not practice of cervical cancer screening was feeling healthy.

**Conclusion and recommendation:**

This study identified that the knowledge status of female police commissioners were poor and practice of cervical cancer screening were found to be very low. Some of the factors associated with the cervical cancer screening practice were age, marital status, monthly income and knowledge of the women. In addition, feeling being health was associated poor practice of cervical cancer screening. To alleviate this problem the health authorities at different level of the health system should take massive awareness creation activities through various communication channels about screening service prepare screening campaign.

**Supplementary Information:**

The online version contains supplementary material available at 10.1186/s12885-023-11478-x.

## Introduction

Cervical cancer is a cancer of the lower-most part of the uterus [1]. Cervical cancer is the second most common cancer which accounts for 22% of all female cancers and 12% of all newly diagnosed cancers every year, and the leading cause of cancer death in African women [1].

Cervix cancer is a progressive disease originating from abnormal cervical dysplasia, which gradually progresses to cervical cancer. It is a major public health issue and a leading cause of death among women worldwide. As the fourth most frequent cancer in women, it had an estimated 570,000 cases in 2018 [2–4]. Cervical cancer is a leading cause of death in women and the second-leading cause of female cancer-related deaths. Globally, over 2.7 million women, 85% of whom live in low- and middle-income countries, are at risk of acquiring cervical cancer [3]. Human papilloma virus (HPV), a sexually transmitted infection, can lead to cervical intraepithelial neoplasia and cervical cancer. Women with multiple sexual partners, those with multiple sexual consorts, or those who have been previously exposed to the virus are at higher risk for cervical cancer [5]. Cervix cancer is the second most common cancer among women globally, with 500,000 new cases diagnosed and over 250,000 deaths annually. Cervical cancer is a global health concern, with 80,000 women aged 15 and older at risk each year of being diagnosed with it. In low- and middle-income countries (LMICs), including Ethiopia, cervical cancer is the most common cancer affecting reproductive organs and the leading cause of death among women. In Ethiopia, cervical cancer ranks as the most frequent cancer among women, causing 4732 deaths annually. Ethiopia has a 35.9 incidence and mortality rate, with 7,600 cases and 6,000 deaths annually [6–9]). Early diagnosis and treatment are crucial to reducing the disease's incidence and mortality [8].

Cervical cancer screening detects abnormal cervical cells, including precancerous lesions, and early-stage cervical cancer, reducing incidence and mortality. Worldwide cervical cancer screening programs and the introduction of the Papanicolaou (Pap) test have significantly reduced mortality and morbidity in developed countries. In Ethiopia, screening is mostly conducted when a woman presents with symptoms. However, limited studies exist on knowledge and practice of cervical cancer screening in Ethiopia [1, 8, 10].

The Federal Ministry of Health Ethiopia (FMOH) Guideline recommends screening for cervical cancer in women aged 25–65, who have never had a Pap smear, had abnormal bleeding, or had cervix abnormalities. Over 80% of cervical cancers in sub-Saharan Africa are detected late, due to lack of information and lack of prevention services. This late stage is associated with low survival rates and limited treatment options in low-resource countries like Ethiopia [6].

Cervical cancer is prevalent in sub-Saharan Africa, with low survival rates and limited information. Low awareness, ineffective screening programs, and insufficient women's health attention contribute to the higher incidence rate [11].

Knowledge about cancer of the cervix and its screening is important in screening uptake. Women with low levels of knowledge about cervical cancer and its prevention are less likely to access screening services [5]. Early diagnosis and treatment are crucial to reducing the disease's incidence and mortality [8]. This study aimed to assess cervical cancer screening knowledge and practice among women police commissioners in Addis Ababa, Ethiopia. focusing on the impact of low awareness on screening uptake. The research aims to improve access to screening services and identify associated factors.

## Method and materials

### Study setting, study design and period

An institutional-based cross-sectional study was conducted at the Addis Ababa police commission in Lidet Sub-city police department from December 1st to January 30th, 2022 in Addis Ababa, Ethiopia. The Addis Ababa police commission was re-established in 1983 by the Council of Ministers. Without prejudice to its legal and professional independence, the commission shall be accountable to the Federal Police Commission. The federal police commission determines and follows up on the implementation of the commission's organization, procedures, training, policies on crime prevention and investigation, direction of strategy, and standardization. The Addis Abeba Police Commission has 20,000 officers, of whom 5502 are female police officers. Addis Ababa has 10 police departments, 45police stations, and 12 police clinics provide services for police members.

### Sample size determination

The sample size for cervical cancer screening was calculated using a single population proportion formula with a 95% confidence level, 5% margin of error, and 42.7% population proportion [12]. The total sample consisted of 376 female police officers. The required sample size was determined using Epi software, considering factors related to knowledge and practice. The study findings were analyzed from (Table 1).

**Table 1:** Sample size calculation with different variables factors associated with Knowledge / Practice of cervical cancer screening

I. No	Factors associated with Practice of cervical cancer screening	Ratio	AOR	Sample Size	Non Response Rate	Final Sample size	Reference
1	Attitude towards cervical cancer screening	1.1	3.023	352	17.6	370	[9]
2	Work place of the respondents	1.1	3.424	272	13.6	286	

The calculated sample size for both objectives and maximum sample size was taken for the final required sample size. The final total sample size calculated was 370.

#### Sampling procedure

The study was conducted at the one sub-city of the Lideta Police Department in the commission. Samples were selected by simple randomness from women police members working at the Lideta police department.

### Data collection tools, and quality control method

The questionnaire was designed to include all information such as sociodemographic characteristics, knowledge, attitude, and practice of cervical cancer screening was adopted from validated published article [13]. A study assessed participants' knowledge of cervical cancer by listing questions about risk factors, sexual partners, early sexual intercourse, HPV infection, cigarette smoking, and vulnerable factors in women. The five-item scale was dichotomized, with "Yes" being 1 and "No/Don't know" being 0. A total knowledge score of 20 was computed, categorized as poor knowledge (0–9), and good knowledge (10–20). Participants were asked to choose between "Yes" or "No" [13].

The study assessed participants' attitudes on the prevalence of cervical carcinoma, its transmission, prevention, and cost of cervical cancer screening. They were asked to rate each statement on a 5-point Likert scale, with respondents choosing options like "strongly agree," "agree," "neither agree nor disagree," "disagree," or "strongly disagree." The results were combined for easy presentation, responses for “strongly agree” and “agree” and for “disagree” and “strongly disagree” were combined [13].

The study assessed participants' cervical cancer screening practices by asking questions about the Pap smear test and its usefulness for early detection. Participants were asked about their experience with Pap smear testing, its interval, steps for abnormalities, and reasons not to undergo it. The questionnaire also inquired about the best time, who should administer the test, the procedure, and its benefits. Participants were also asked about their perception of Pap smear testing as a good practice and its perceived pain. Additionally, they were asked about their awareness of HPV vaccination in their hospital and whether they had received it [13]. The adopted questionnaires were prepared in English and translated to Amharic for language barriers, and then back to English for consistency. The data were collected through a structured, self-administered questionnaire from 361 randomly selected female police officers by exempting women who were not members, had been hired for less than six months, or were severely ill during data collection period.

The questionnaire was pretested on 5% of the sample population, not part of the study group, in non-selected sub-cities. Corrections were made after the pre-test. Field questionnaires were checked for completeness, missed values, and unlikely responses daily. Amendments were made before data collection. Data clean-up and cross-checking were conducted before analysis.

### Data processing and analysis

The collected data was coded, entered and analyzed using SPSS Version 26 for Windows. It was checked for completeness, cleaned, processed and analyzed accordingly. Descriptive statistics used to present study participants’ characteristics and fitted a binary logistic regression model to identify factors associated with Knowledge and Practice of cervical cancer screening. Bivariable analysis was conducted to identify explanatory variables, and all explanatory variables with a *p*-value of less than 0.25 were included in the multivariable logistic regression analysis. Model fitness was checked using Hosmer and Lemeshow goodness of fit, and multicollinearity was assessed using the variance inflation factor (VIF). The level of significance was determined using adjusted odds ratio and *P*-value of less than 0.05.

### Operational definition and terms

#### Cervix: an opening of the uterus

Cancer: a disease caused by an uncontrolled division of abnormal cells in a part of the body.

Cancer screening is a procedure that is performed to identify the presence of an abnormal cell in a particular tissue.

Good knowledge of cervical cancer screening: women who scored of total knowledge score of 20 was computed, categorized as poor knowledge (0–9), and good knowledge (10–20). Participants were asked to choose between "Yes" or "No" [13].

Women who had responses for “strongly agree” and “agree” were combined and considered to have a positive or favorable attitude, and for “disagree” and “strongly disagree” were combined considered to have a negative or unfavorable attitude toward cervical cancer screening [13].

Cervical cancer screening refers to women who have previously been screened for cervical cancer, which is represented by yes (1), and those who have never been screened, which is represented by no (0).

## Results

### Socio-demographic characteristics

The complete response rate was 97.57% (361/371). This found that 59.5% of the total study participants were aged between 18 and 29 years old, and 47.3% were married in marital status. The majority were married, with 47.3%) being married. The majority were diploma holders, with 51.1% participating in orthodox religion. The income per month was greater than or equal to 5251 Ethiopian birr. see details in Table 2.

**Table 2:** Frequency Socio-demographic characteristics of participants Addis Ababa police commission Ethiopia, Jan 2022(361)

Variable	Categories	Frequency	Percent
Age	18–29	220	59.5
	30–40	107	28.9
	> 40	28	7.6
The most frequent age of participants	18–29		
Educational status	Grade10-12	87	23.5
	Certificate	74	20.0
	Diploma	164	44.3
	Degree and above	33	8.9
Marital status	Single	149	40.3
	Married	175	47.3
	Divorced	19	5.1
	Widowed	16	4.3
Religion	Orthodox	192	51.9
	Muslim	32	8.6
	Protestant	107	28.9
	Other	26	7.0
Monthly Income	1900–3200	87	23.5
	3201–5250	90	24.3
	> = 5251	175	47.3
Mean salary	Mean = 2.25(3201–5251)		

### Knowledge of respondents about cervical cancer screening

All study participants had heard about cervical cancer, and the primary source of information (66.5%) came from health professionals. 115 (31.1%) people said a virus causes cervical cancer. The majority of participants (226, or 61.1%) said cervical cancer is a transmitted disease; 164, or 44.3%, said sexual intercourse is a way of transition; and 93, or 25%, said they didn't know the method of transmission. 146 (39.5%) participants mentioned that all foul-smelling virginal discharge, irregular vaginal bleeding, and postcoital bleeding are symptoms of cervical cancer. The majority of participants know the risk of cervical cancer; of them, 114 (30.8%) answered all the following (having multiple sexual partners, early sexual intercourse, cigarette smoking, and infection with the human papilloma virus) are the risks for cervical cancer. The majority of participants said all of the following are preventive methods: avoiding multiple sexual partners, avoiding early sexual intercourse, HPV vaccination, and quitting smoking, while 149 (40.3%) were unaware of cervical cancer treatment. 152 (41.1%) of participants did not know the frequency of cervical cancer screening, and 176 (47.6%) said all women should be screened, followed by 76 (20.5%) who said women over the age of 25 should be screened see details in Table 3.

**Table 3:** Frequency of Knowledge variable of the study participants Addis Ababa police commission Ethiopia, 2022( *n* = 361)

Variable	Category	Frequency	Percent
Have you heard about cervical cancer?	Yes	361	100
	No	0	0
If yes for number 1 what is your source	Friend	29	7.8
	Health professional	246	66.5
	Radio	26	7.0
	TV	49	13.2
What is the causative agent of cervical cancer?	virus	115	31.1
	Bacteria	65	17.6
	Fungus	34	9.2
	Parasite	33	8.9
	I don’t know	111	30.0
Think cervical cancer transmitted disease?	Yes	226	61.1
	No	134	36.2
If the answer Q4 is yes, how cervical cancer transmit	sexual intercourse	164	44.3
	mother to child	25	6.8
	air born	6	1.6
	I don’t know	93	25.1
What are the symptoms of cervical cancer?	foul smell Vaginal discharge	72	19.5
	irregular vaginal bleeding	30	8.1
	post coital bleeding	35	9.5
	all of the above	146	39.5
	I don’t know	75	20.3
know the risk factor of cervical cancer?	Yes	260	70.3
	No	96	25.9
If yes for question 5 what are those	Having multiple sexual partner	97	26.2
	Early sexual intercourse	35	9.5
	Cigarette smoking	20	5.4
	Infection by human pailoma virus	16	4.3
	All of the above	114	30.8
How can prevent cervical cancer	Avoid multiple sexual partner	53	14.3
	Avoid early sexual intercourse	22	5.9
	Human papilloma virus vaccination	21	5.7
	Quit cigarette smoking	16	4.3
	All the above	183	49.5
	I don’t know	60	16.2
know the treatment of cervical cancer?	Surgery	22	5.9
	Chemotherapy	29	7.8
	Radiotherapy	59	15.9
	All of the above	94	25.4
	I don’t know	149	40.3
know the Screening frequency?	Once in a year	87	23.5
	Every three year	56	15.1
	Every five year	58	15.7
	I don’t know	152	41.1
Who should be screened for cervical cancer	Women age is greater than 25 years	76	20.5
	Prostitute	47	12.7
	All women	176	47.6
	I don’t know	56	15.1
Knowledge of cervical cancer	Good Knowledge	183	49.5
	Poor knowledge	187	50.5

**Fig. 1:**
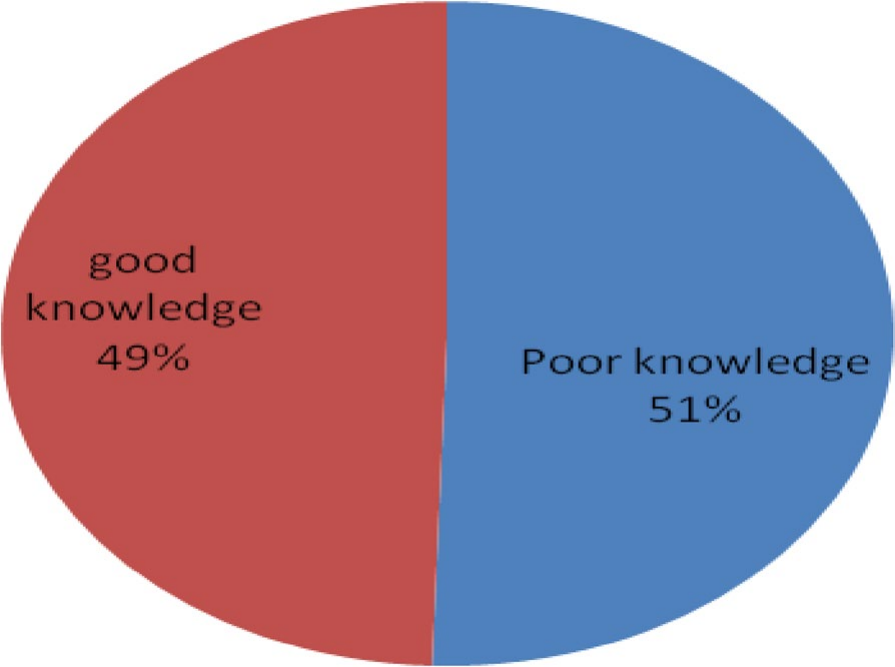
Knowledge of cervical cancer participants in Addis Ababa police commission, Ethiopia, 2022

This chart indicates the prevalence of good knowledge on cervical cancer screening, 183(49.5%) with 95% CI (44.3–54.43) see details in Fig. 1.

### Attitude of participants towards cervical cancer screening

Early identification of cervical cancer was beneficial to 208 (56.2%) of the participants.

According to these 175 (47.3%) participants, 95.2% feel they have a possibility of having cervical cancer, and the majority also believe there are efficient measures to lessen the danger of cervical cancer. Cervical cancer is the leading cause of death for the majority of participants (40.8%).

On the topic of whether any women had cervical cancer, 113 (30.5%) individuals were undecided. The majority of participants (40.8%) strongly believe that cervical cancer is the leading cause of mortality. The majority of participants (111/30.0%) agreed that cervical cancer can be cured, and 116 (31.4%) agreed that screening can help prevent cervical cancer. A total of 149 women (40.3%) volunteered to get tested for cervical cancer see Table 4.

**Table 4:** Frequency of Attitude of participants towards cervical cancer screening Addis Ababa police commission, Ethiopia, Jan, 2022(*n* = 361)

Variable	Categories	Frequency	Percent
Helpful to detect cervical cancer early	Strongly agree	208	56.2
	Agree	75	20.3
	Neutral	57	15.4
	Disagree	7	1.9
	Strongly disagree	8	2.2
Believe that you have the chance of getting cervical cancer?	Strongly agree	71	19.2
	Agree	95	25.7
	Neutral	87	23.5
	Disagree	73	19.7
	Strongly disagree	29	7.8
Believe that getting cervical cancer is serious for you?	Strongly agree	175	47.3
	Agree	96	25.9
	Neutral	65	17.6
	Disagree	11	3.0
	Strongly disagree	6	1.6
Think that there are effective methods to reducing the risk of seriousness of cervical cancer?	Strongly agree	126	34.1
	Disagree	104	28.1
	Neutral	102	27.6
	Disagree	18	4.9
	Strongly disagree	4	1.1
Think cancer of the cervix is the cause of death?	Strongly agree	151	40.8
	Agree	92	24.9
	Neutral	68	18.4
	Disagree	28	7.6
	Strongly disagree	13	3.5
Think any women acquired cervical cancer?	Strongly agree	91	24.6
	Agree	78	21.1
	Neutral	113	30.5
	Disagree	53	14.3
	Strongly disagree	17	4.6
Think cervical cancer can be treated?	Strongly agree	96	25.9
	Agree	111	30.0
	Neutral	95	25.7
	Disagree	37	10.0
	Strongly disagree	15	4.1
Think screening helps in prevention of cervical cancer?	Strongly agree	116	31.4
	Agree	128	34.6
	Neutral	78	21.1
	Disagree	15	4.1
	Strongly disagree	16	4.3
Willing to be screened?	Strongly agree	149	40.3
	Agree	114	30.8
	Neutral	59	15.9
	Disagree	15	4.1
	Strongly disagree	17	4.6

**Fig. 2:**
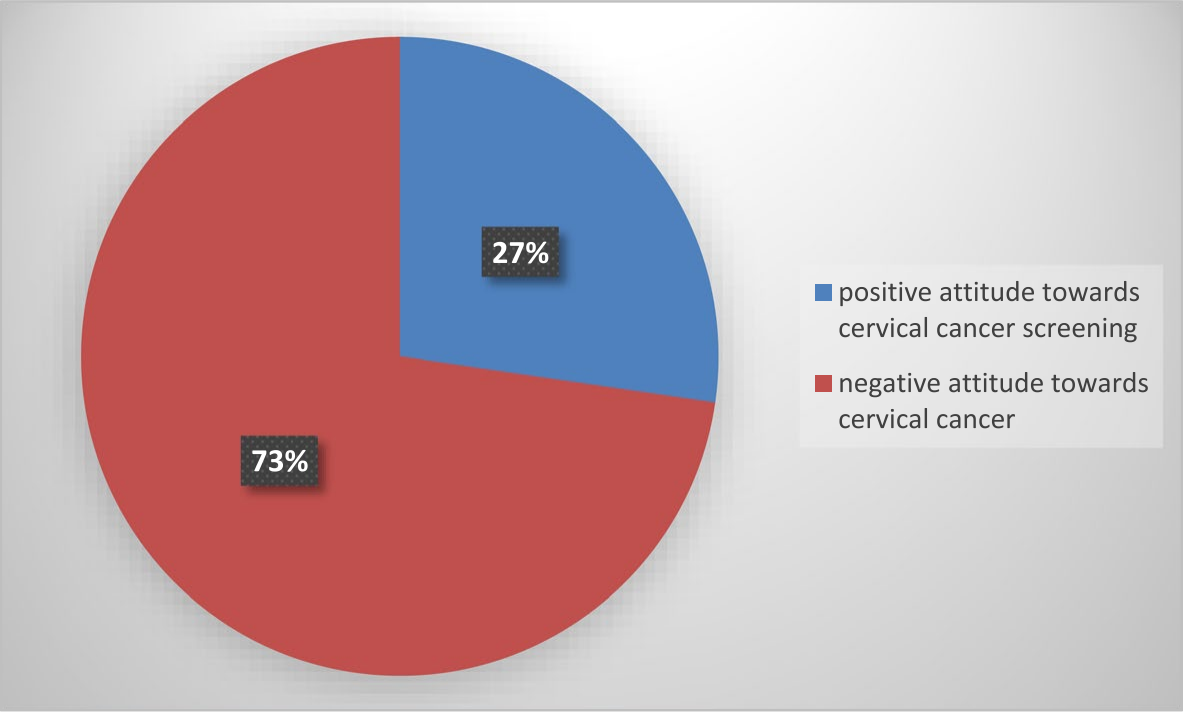
Magnitudes of positive attitude towards cervical cancer screening among study participants in Addis Ababa police commission, Ethiopia, 2022

This study found that indicates the magnitudes of positive attitude towards cervical cancer screening, 101(27.3%), while the majority 269 (72.7%) had negative attitude towards cervical cancer screening and vaccination. see details in Fig. 2.

### Cervical cancer screening practices

The majority of participants (296, or 80%) practice sexual intercourse; among them, 209 (56%), who started sexual intercourse at an age greater than or equal to 18 years, had a single sexual partner. Of the total study participants, 293 (81.6%) had not been screened for cervical cancer; the majority of 114 (30.8%) participants reasons for not being screened were perceived as healthy. Almost all 348 (94.1%) of the participants were not vaccinated for cervical cancer; see details in Table 5.

**Table 5:** Frequency of cervical cancer screening practice of the participants Addis Ababa police commission, Ethiopia 2022. (*n* = 361)

Variable	Categories	Frequency	Percent
Practice sexual intercourse	Yes	296	84.1
	No	56	15.9
Age of 1^st^ sexual intercourse	< 18	83	22.4
	≥ 18	209	56.5
Number of sexual partners	Single	194	52.4
	Multiple	104	28.1
Ever screened for cervical cancer	Yes	68	18.4
	No	293	81. 6
Major reason for not screened	I am healthy	114	30.8
	It may be painful	45	12.2
	I have no interest	45	12.2
	Never informed about screening of cervical cancer	46	12.4
	other reason	63	17.0
vaccinated for cervical cancer	Yes	2	0.5
	No	348	94.1

**Fig. 3:**
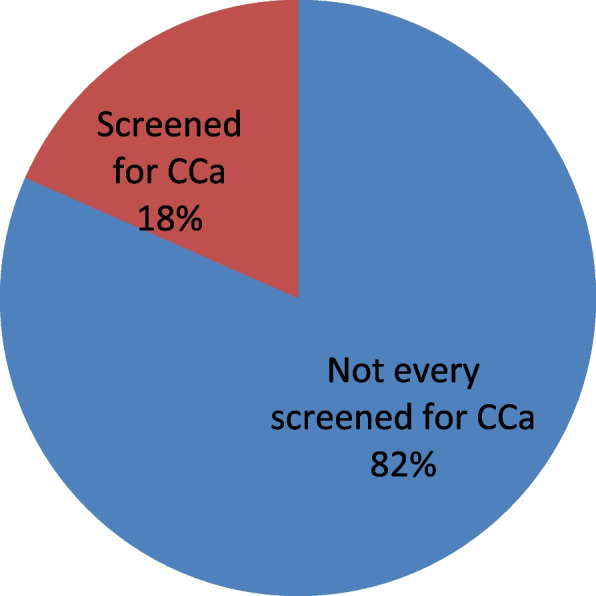
Practice of cervical cancer screening participants of Addis Ababa police commussion Addis Ethiopia 2022

This Fig. 3 indicates the prevalence of cervical cancer screening was 68 (18.4%) with a 95% CI of 14.3–22.4. The main reasons for not being screened for cervical cancer 114 (30.8%) feel healthy, 45 (12.2%) think the procedure may be painful, 45 (12.2%) have no interest, 46 (12.4%) were never informed about screening for cervical cancer, and 63 (17.0%) have other reasons for not screening (Fig. 3).

#### Factors associated with knowledge towards cervical cancer screening

A binary logistic regression was used to examine the relationship between cervical cancer screening knowledge and prevalence, and it was discovered that monthly income, attitude, sexual intercourse practice, and knowledge of cervical cancer screening were all significantly related.

As shown in Table 6, participants with a positive attitude were 2.03 times more likely than those with a negative attitude to have good knowledge about cervical cancer screening [AOR = 2.03, 95% CI: (1.247–3.305)], and participants who had screened for cervical cancer were more likely to have good knowledge about cervical cancer screening than those who had not screened [AOR = 2.01, 95% CI: (1.145–3.53)].

**Table 6:** Multivariate analysis of knowledge of cervical cancer screening (*n* = 361)

Variable	Categories	Knowledge of cervical cancer screening		AOR	*P* value
		Poor knowledge	Good knowledge		
Attitude	Negative attitude	64(63.4%)	37(36.6%)	1	
	positive attitude	114(43.82%)	146(56.2%)	2.030(1.247–3.305)	0.004
Practice for ccs	No	154(52.6)	139(47.4%)	1	
	Yes	24(35.5%)	44(64.7%)	2.011(1.145–3.53)	0.015

#### Factors associated with practice towards cervical cancer screening

Table 7 showed that age, educational status, marital status, religion, monthly income, and knowledge had a significantly positive association with the practice of cervical cancer screening at the binary level and were eligible for multiple logistic regression analysis for the identified factors.

**Table 7:** Multivariate analysis of practice of cervical cancer screening (*n* = 361)

Variables	Categories	Screened for cervical cancer		AOR	*P* value
		Yes	No		
Age	18–29	27(12.6%)	187(87.4%)	1	
	30–40	24(22.9%)	81(77.1%)	0.556(0.242–1.277)	0.166
	> 40	17(60.7%)	11(39.3%)	3.240(1.077–9.749)	0.036
Marital status	Single	7(4.9%)	135(95.1%)	1	
	Married	47(27.0%)	127(73.0%)	3.884(1.550–9.732)	0.004
	Divorced	6(31.6%)	13(68.4%)	3.605(.886–14.659)	0.073
	Widowed	8(53.3%)	7(46.7%)	14.453(3.11–67.173)	0.001
Religion	Orthodox	48(25.3%)	142(74.7%)	1	
	Muslim	4(13.3%)	26(86.7%)	8.654(1.647–45.463)	0.011
	Protestant	14(13.9%)	87(86.1%)	2.049(.250–16.757)	0.504
	Others	2(7.7%)	24(92.3%)	4.722(.830–26.875)	0.080
Monthly income	1900–3200	4(4.8%)	80(95.2%)	1	
	3201–5250	10(11.2%)	79(88.8%)	1.860(.518–6.673)	0.341
	> = 5251	53(30.5%)	121(69.5%)	4.820(1.441–16.118)	0.011
Knowledge	Poor knowledge	24(13.5%)	139(76.0%)	1	
	Good knowledge	44(24.0%)	154(86.5%)	2.345(1.216–4.52)	0.011

The age group > 40 years was 3.2 times more likely to be screened than the age group 18–29 years [AOR = 3.240, 95% CI: 1.077–9.749)].

Religion Muslims are 8.65 times more likely than orthodox to be screened [AOR = 8.654, 95%CI, (1.647–45.463)]. Marital status: married women were 3.884 times more likely than single women to be screened for cervical cancer [AOR = 3.884, 95% CI: 1.550–9.732], and a monthly salary of more than 5251 BR 4.820 times more likely to be screened than those earning 1900–3200 BR per month [AOR = 4.82, 95% CI: (1.441–16.118)], and those who were well-informed were 2.345 times more likely to be screened for cervical cancer [AOR = 2.345, 95% CI: 1.216–4.52]; these factors were independent predictors of cervical cancer screening among policewomen.

## Discussion

Cervical cancer screening enables the detection of abnormal cervical cells, including precancerous cervical lesions, as well as early-stage cervical cancer. Routine cervical screening has been shown to reduce both the incidence and mortality of the disease. This study showed all participants heard about cervical cancer, 49.5% had good knowledge about cervical cancer screening, and 18.4% had good screening practices. Associated factors that affected knowledge were participants' monthly income, attitude, practice of sexual intercourse, and screening practices. Participants' age, educational status, marital status, monthly income, religion, and knowledge were associated factors for cervical cancer screening practice; the main reason they did not get screened was that they felt healthy. When compared to previous studies conducted globally, the knowledge of the participants in this study is higher; 42.22% were aware of the screening [14]. Further, the study conducted in Nepal 12.6% and Tanzania17.3% discovered adequate knowledge [15, 16].

A study conducted in Saudi Arabia 4.0% had good of knowledge of cervical cancer [16] and a systematic review conducted Ethiopia reported that 47.16% had knowledge about the screening [17] and Previous studies conducted in various parts of Ethiopia among reproductive age women with different background has shown revealed in Adigrat town, Northern Ethiopia in 2019,46.4%, of participants had a knowledge on cervical cancer [7] besides in the rural Butajira and Batu36% and 19.7% of women participants had good knowledge level on cervical cancer respectively [18, 19]. In Addis Ababa’s studies taken on reproductive health service, 43.8% of women had knowledge of cervical cancer [8]. Further, in 2015 with in Addis Ababa, 27.7% women had adequate knowledge of cervical cancer screening [8]. On the other hand, on urban health extension workers studied, 48.4% had good knowledge about cervical cancer screening [12]. The difference may be due to access for health facilities and availability of technology, time gap of the study, and different in educational statue of participants.

on other hand this study lower than the study at University of Gondar,more than half of the respondents 59.3% had a good knowledge [18]. The difference might be due to study area difference, time of study, educational status and participants’ difference. Participants with a positive attitude were 2.03 times more likely to have good knowledge about cervical cancer screening than those with a negative attitude. Those who had been screened for cervical cancer were more likely than those who had not. Regarding the practice of cervical cancer the study showed 18.4% participants screened for cervical cancer this is greater the previous studies done among women globally 13.26% [12], in Nepal and Tanzania 13.6% and 14.3% respectively [15, 16], Ethiopia 2.9% [8] and study in Addis Ababa 3.5% [8]. The difference is due to time, location difference, availability of health facility and availability of screening service near to participant and knowledge of participants. This study is less cervical screening with the study systematic review and meta-analysis in Ethiopia which was 26.15% [17], Aribaminch 27.7% [2] and other study done in Addis Ababa 25% [8]. This is the gap in knowledge, study setting time and study area difference. And the finding near similar with the studies done in Nigeria 20.6% [5] and Sidama 17.8% [20]. The age group > 40 years was 3.2 times more likely to be screened for cervical cancer than the age group 18–29 years. Religion Muslims were 8.65 times more likely than orthodox to be screened. Marital status was 3.884 times more likely for married women, monthly earnings greater than 5251 billion were 4.820 times more likely, and those who were well-informed were 2.345 times more likely, these supported with previous study done [21]. This study aimed to assess the knowledge and Practice of cervical cancer screening and associated factors among women police members in Addis Ababa. police commission. Appropriate sampling techniques were used, but the main limitation of the study was that it was conducted among Police and used self-report instruments. The methodology of relying on respondents self-reporting also has challenges of data bias and inconsistency.

### Clinical implication

This study offers valuable information on cervical cancer awareness, highlighting the need for trained reproductive health professionals and improved access to cervical screening. The findings can be used in local policy making to establish better medical awareness facilities in the Addis Ababa region and beyond. Additionally, information can be circulated among females using pamphlets in places frequented by women, such as female restrooms.

## Conclusion

According to the findings of this study, all participants had heard of cervical cancer, and half of the participants had good knowledge of cervical cancer screening but little experience with screening for cervical cancer. Health professionals were the main source of information. Monthly income, attitude, and practice of cervical cancer screening were associated with good knowledge. Participants' age, marital status, religion, monthly income, and knowledge are associated with practice screening. Participants' feelings that they were healthy were the perceived reasons for the low screening practice.

The findings suggest that further awareness is necessary to improve the knowledge and practice of cervical cancer screening. To do this, awareness-creation activities should be undertaken by the Addis Ababa Police Commission health department with a corporate federal police hospital. Health professionals should educate women about the benefits and risk factors for cervical cancer, recommend awareness campaigns to encourage eligible women to get screened, inform women of the availability of the service when they go to a health facility for other services. Reproductive health specialists should pay attention to screening women who came to get other reproductive health services, train health professionals to give screening services, and prepare cervical cancer screening campaigns for vulnerable women. Health extension workers should focus on educating the community and advocating for eligible women to be screened.

Policymakers and programmers should strengthen the existing screening program, and an NGO working in this field should assist with fund-raising for health professional training and screening facilities. To alleviate this problem, health authorities at different levels of the health system should undertake massive awareness-creation and provide screening services.

### Supplementary Information

**Additional file 1: Part one.** Socio-demography characteristics. Circle on your answer. **Part 2.** Knowledge Questions. **Part 3.** Practice question towards cervical cancer screening. **Part 4.** Attitude questions towards cervical cancer screening.

## Data Availability

Datasets used in the current study are available from the corresponding author upon reasonable request.

## Abbreviations

A.A: Addis Ababa
AIDS: Acquired Immune Deficiency Syndrome
AOR: Adjusted odd Ratio
COR: Crude Odd Ratio
CI: Confidence Interval
FMOH: Federal Ministry of Health Ethiopia
HIV: Human Immune Virus
HPV: Human papilloma virus
SPSS: Statistical Package for Social Science
STD: Sexually Transmitted Disease
STI: Sexually Transmitted Infection
WHO: World Health Organization
