# Supplementary material for: Knowledge, practice of cervical cancer screening and associated factors among women police members of Addis Ababa police commission Ethiopia
Source: BMC Cancer. 2023 Oct 10;23:961. doi: 10.1186/s12885-023-11478-x (PMC10566075; doi:10.1186/s12885-023-11478-x)
Supplement: Supplementary file 1 — Additional file 1: Part one. Socio-demography characteristics. Circle on your answer. Part 2. Knowledge Questions. Part 3. Practice question towards cervical cancer screening. Part 4. Attitude questions towards cervical cancer screening. [file 12885_2023_11478_MOESM1_ESM.docx]

**Supplementary Information: Structured English version questionnaire**

**Part one: Socio-demography characteristics. Circle on your answer**

| **S.no.** | **Questions** | **Response** | **Remark** |
| --- | --- | --- | --- |
| 1 | Age | 18-29  30-40  >40 | 1  2  3 |
| 2 | Education status | Grade10-12 Certificate  Diploma  Degree and above | 1  2  3  4 |
| 3 | Marital status | Single  Married  Divorced  Widowed | 1  2  3  4 |
| 4 | Religion | Orthodox  Muslim  Protestant  Others | 1  2  3  4 |
| 6 | Monthly Salary | 1900-3200  3201-5250  ≥5251 | 1  2  3 |

**Part 2 Knowledge Questions**

| **S.no** | **Questions** | **Response** | **Remark** |
| --- | --- | --- | --- |
| 1 | Have you heard about cervical cancer? | Yes  No | 1  2 |
| 2 | If yes for number 1 what is your source | Friend  Health professional  Radio  TV | 1  2  3  4 |
| 3 | What is the causative agent of cervical cancer? | virus  bacteria  fungus  parasite  I don’t know | 1  2  3  4  5 |
| 4 | Do you think cervical cancer transmitted disease? | Yes  No | 1  2 |
| 5 | If the answer Q4 is yes, how cervical cancer transmit | sexual intercourse  mother to child  air born  I don’t know | 1  2  3  4 |
| 6 | What are the symptoms of cervical cancer? | foul smell Vaginal discharge  irregular vaginal bleeding  post coital bleeding  all of the above  I don’t know | 1  2  3  4  5 |
| 7 | Do you know the risk factor of cervical cancer? | Yes  No | 1  2 |
| 8 | If yes for question 5 what are those | Having multiple sexual partner  Early sexual intercourse  Cigarette smoking  Infection by human pailoma virus  All of the above | 1  2  3  4  5 |
| 9 | How can prevent cervical cancer | Avoid multiple sexual partner  Avoid early sexual intercourse  Human papilloma virus vaccination  Quit cigarette smoking  All the above  I don’t know | 1  2  3  4  5  6 |
| 10 | Do you know the treatment of cervical cancer? | Surgery  Chemotherapy  Radiotherapy  All of the above  I don’t know | 1  2  3  4  5 |
| 11 | Do you know the Screening frequency? | Once in a year  Every three year  Every five year  I don’t know | 1  2  3  4 |
| 12 | Who should be screened for cervical cancer | Women age is greater than 25 years  Prostitute  All women  I don’t know | 1  2  3  4 |

Part3 **Practice question towards cervical cancer screening**

| **S.no** | **Questions** | **Response** | **Remark** |
| --- | --- | --- | --- |
| 1 | Have you ever practice sexual intercourse? | Yes  No | 1  2 |
| 2 | At what age you start sexual intercourse? | <18  ≥18 | 1  2 |
| 3 | How many sexual partners do you have till now? | Single  Multiple | 1  2 |
| 4 | Have you ever screened for cervical cancer? | Yes  No | 1  2 |
| 5 | If the answer is no for question no.4 what is the reason for not screened? | I am healthy  It may be painful  I have no interest  Never informed about screening  of cervical cancer  other reason | 1  2  3  4  5 |
| 6 | Are you vaccinated for cervical cancer? | Yes  No | 1  2 |

**Part 4: Attitude questions towards cervical cancer screening**

| **S.No** | **Questions** | **Response** | **Remark** |
| --- | --- | --- | --- |
| 1 | Do you think it is helpful to detect cervical cancer early? | Strongly agree  Agree  Neutral  Disagree  Strongly disagree | 1  2  3  4  5 |
| 2 | Do you believe that you have the chance of getting cervical cancer? | Strongly agree  Agree  Neutral  Disagree  Strongly disagree | 1  2  3  4  5 |
| 3 | Did you believe that getting cervical cancer is serious for you? | Strongly agree  Agree  Neutral  Disagree  Strongly disagree | 1  2  3  4  5 |
| 4 | Do you think that there are effective methods to reducing the risk of seriousness of cervical cancer? | Strongly agree  Agree  Neutral  Disagree  Strongly disagree | 1  2  3  4  5 |
| 5 | Do you think cancer of the cervix is the cause of death? | Strongly agree  Agree  Neutral  Disagree  Strongly disagree | 1  2  3  4  5 |
| 6 | Do you think any women acquired cervical cancer? | Strongly agree  Agree  Neutral  Disagree  Strongly disagree | 1  2  3  4  5 |
| 7 | Do you think cervical cancer can be treated? | Strongly agree  Agree  Neutral  Disagree  Strongly disagree | 1  2  3  4  5 |
| 8 | Do you think screening helps in prevention of cervical cancer? | Strongly agree  Agree  Neutral  Disagree  Strongly disagree | 1  2  3  4  5 |
| 9 | Are you willing to screen to cervical cancer? | Strongly agree  Agree  Neutral  Disagree  Strongly disagree | 1  2  3  4  5 |
